# The effect of nanomicelle curcumin, sorafenib, and combination of the two on the cyclin D1 gene expression of the hepatocellular carcinoma cell line (HUH7)

**DOI:** 10.22038/ijbms.2019.35808.8530

**Published:** 2019-10

**Authors:** Sare Hosseini, Jamshidkhan Chamani, Mohadeseh Sinichi, Amir Mohammad Bonakdar, Zeinolabedin Azad, Najmeh Ahangari, Hamid Reza Rahimi

**Affiliations:** 1 Cancer Research Center, Mashhad University of Medical Sciences, Mashhad, Iran; 2 Department of Biology, Faculty of Sciences, Islamic Azad University-Mashhad Branch, Mashhad, Iran; 3 Student Research Committee, School of Para Medicine, Mashhad University of Medical Sciences, Mashhad, Iran; 4 Department of Modern Sciences & Technologies, Faculty of Medicine, Mashhad University of Medical Sciences, Mashhad, Iran; 5 Vascular and Endovascular Surgery Research Center, Mashhad University of Medical Sciences, Mashhad, Iran

**Keywords:** Cyclin D1 Gene, Curcumin, Hepatocellular carcinoma Nanomicelle, Sorafenib

## Abstract

**Objective(s):**

Hepatocellular carcinoma (HCC) is one of the most significant health condition around the world. As the only curative therapies, liver transplantation and surgical resection are the clinical treatments of HCC. Due to the systemic toxicity and severe side effects of these treatments, it is vital to establish new therapeutic approaches. The present study aimed to compare cyclin D1 (CCN D1) gene expression in hepatocellular carcinoma cell line (HUH7) when it is treated with nanomicelle curcumin and sorafenib. The purpose was to identify toxicity risk and antioxidant activity of these drugs.

**Materials and Methods:**

The toxic dose (IC_50_) of nanomicelle curcumin and sorafenib were detected after treatment of HUH7 cell lines with different dose of mentioned agents followed by MTT assay. CCN D1 gene expression was evaluated using real-time PCR. Following the Tukey’s multiple comparison tests, statistical analysis is done through Student’s t-test or ANOVA.

**Results:**

The expression of the CCN D1 gene was statistically significant (*P*<0.001) at 289.31, 128 and 152.36 for sorafenib, nanomicelle curcumin and SNC (sorafenib-nanomicelle curcumin) respectively. The finding of this study revealed that, in comparison to sorafenib alone, the treatment of HUH7 with a nanomicelle curcumin IC_50_ dose, in combination with sorafenib, might down-regulate CCN D1 gene expression.

**Conclusion:**

The present research indicates that the treatment of the cell line with only nanomicelle curcumin results in the down-regulation of cyclin D1. To further decrease cyclin D1 expression, the co-delivery of curcumin and sorafenib appears to induce the apoptotic process. As a result, the effect of sorafenib cytotoxicity and CCN D1 gene expression decreases twofold.

## Introduction

As one of the most prevalent health conditions, hepatocellular carcinoma (HCC) accounts for more than 626,000 new cases globally per year. The incidence of HCC is increasing in Europe and the United States as well as in the Asia-Pacific region (1). After lung and then stomach cancers, HCC is the third most frequent cause of deaths from cancer around the world (2). Other than surgery, the clinical treatment of HCC is chemotherapy, yet surgical resection, and liver transplantation are the only curative therapies among the current therapeutic options. However, as most patients are diagnosed in the advanced stages, surgical therapies are not a suitable option. Sorafenib is a nonspeciﬁc multi-kinase inhibitor that has been used in the clinical practice for individuals who are in advanced stages of HCC. But, it merely extends the lifetime of patients from 7.9 to 10.7 months (3, 4). Worse still, after sorafenib failure, there are no other effective replacements among the therapeutic agents. Then, it is crucial to come up with a new way to develop the therapeutic efficiency of sorafenib on HCC (5). Moreover, most anticancer drugs are highly toxic with low specificity, which lead to systemic toxicity and acute side effects. It is needed to improve the tumor targeting drug delivery system to develop targeted therapies for achieving better efficiency with more limited side effects than chemotherapy agents on healthy tissues (6). Nanotechnology in medication, and more specific drug delivery usage is spreading quickly. Remarkably based on pharmaceutical sciences, nanoparticles are being used to limit toxicity and side effects of drugs, but recently, it has been noticed that it is possible for carrier systems themselves to have risks for the patient (7). Targeted nanoparticles have acquired substantial attention as an efficacious drug and gene delivery system, which is because of their ability in accomplishing the highest accumulation of cytotoxic drugs in tumor tissue, and limited side effects (8). Curcumin is a very active component which comes from the root of turmeric (in Farsi it is called Zardchoobeh) (9). Curcumin is a helpful anti-inflammatory for different types of human chronic inflammatory diseases (10). Curcumin is insoluble in water and ether and it dissolves in ethanol, acetone, and dimethyl sulfoxide (DMSO) (11). Nanotechnologies of curcumin presented in various shapes and sizes (12). It Is known that curcumin has a poor oral bioavailability (13). Incorporation of curcumin into micelles can enhance bioavailability up to 185-fold in healthy persons causing no adverse effects (14). According to the studies, the co-delivered nano-assemblies of curcumin and sorafenib have prepared a favorable method to improve the combinational treatment of HCC (5). One of the operative molecules for targeted therapies are cyclins which are pivotal particles in cell cycle control because of their periodic and particular expression through cell cycle progression (15). Recent findings imply that abnormal expression of cyclin D1 probably has a remarkable effect on the growth of human hepatoma as well as other carcinomas. Undeniably, cyclin D1 over-expression is adequate to initiate hepato-carcinogenesis among transgenic mice (16). In this regard, it is vital to establish new therapeutic approaches for these types of tumor. In this study, to identify toxicity risk and antioxidant activity of drugs, we aimed to compare CCND1 gene expression in HUH7 cell line treated with nanomicelle curcumin and sorafenib. 

## Materials and Methods

***Chemicals***

Nano-curcumin was graciously provided by the Exir Nano Sina Company (Tehran, Iran). Each nano-curcumin soft gel contained 80 mg of curcumin. Sorafenib 200 mg pills with tradename Nexavar was obtained from Bayer Schering Pharma (Germany) company.

***Cell lines***

The HUH7 cells (Cat No: C145) were purchased from the Cell Bank, Pasteur Institute of Iran (Tehran, Iran). This cell line in American Type Culture Collection (ATCC) is known as Cell-bance: jcrb0403.

***Cell culture and MTT assay***

The effect of sorafenib, curcumin, and SNC (sorafenib-nanomicelle curcumin) on *in vitro *antiproliferative activities, was measured in HUH7 cells. HUH7 cell lines were cultured in DMEM high glucose (GIBCO, Invitrogen), with 1:100 streptomycin/penicillin and 10% HI-FBS. Summarily, cells were seeded into 96-well plate at 5000 cells/well and were cultured nightlong. After that, curcumin, sorafenib, and SNC were added to each well respectively at predestined concentrations (see Table 1) and incubated for extra 48 hr. Then, the cytotoxicity was measured by MTT (3-(4,5-dimethylthiazol-2-yl)-2,5-diphenyltetrazolium bromide) tetrazolium reduction assay according to standard protocol (5). The cell viability was specified as the absorbance values of samples compared to that of negative controls. Likewise, the half-maximal inhibitory concentration (IC_50_) of every cluster was calculated.

***Cell line preparation before RNA extraction***

To prepare the cell line, at first, one million cells transferred to a 6-well plate. The dose of 7.5 mg/ml nanomicelle curcumin (IC_50_) was added to the first well. A dose of 10 mg/ml sorafenib (IC_50_) was then added to the second well, and also a combination of the IC_50_ dose of nanomicelle curcumin and sorafenib was added to the third well. Then treated 24 hr with the IC_50 _dose of nanomicelle and then 24 hr with the IC_50_ dose of sorafenib, following the procedure 10^6 ^cell from HUH7 cell line with no treating was added to the fourth well as the control sample. The 6-well plate was incubated for 24 hr at 37 ^°^C and 5% concentration of CO_2_.

***Molecular assessment***

RNA extraction has been done using a standard protocol (QIAGEN GmbH, Hilden Germany). Purity and yield of the samples were tested at 260-280 nm with NanoDrop®-1000-Detector (NanoDrop-Technologies, Wilmington, NC). Using the cDNA Synthesis Kit (Parstous Co, cat#5301, Tehran, Iran), One &mgr;g of RNA (1 &mgr;g) was reverse transcribed according to the manufacturers’ instruction. Primers for quantitative real-time polymerase chain reaction are shown in Table 2. Quantitative Real Time-PCR (qRT-PCR) of CCND1 was carried out with the SYBR Green method in an ABI-7900HT sequence detection system (Applied Biosystems, Life Technology, Forster City, CA). Each reaction mixture contained 10 µl of master mix, 1 µl of cDNA, and 10 µl of primer. (3 micro-tube containing synthetized cDNA after treating and one micro-tube as the control sample, containing synthesized cDNA from the untreated cell line.) The quantitative RT-PCR conditions were: 95 ^°^C for 30 sec, 95 ^°^C for 4 sec, then 60 ^°^C for 32 sec, for melting curve: 95 ^°^C for 10 sec, and 60 ^°^C for 60 sec. The 2^-ΔΔCt^ method was utilized to quantify gene expression with glyceraldehyde-3-phosphate dehydrogenase (GAPDH) utilized as a housekeeping gene. Results were expressed as relative fold changes in gene expression and then normalized to the corresponding reference gene (GAPDH) levels (primers in Table 2).

***Statistical analysis***

All measurements were performed in triplicate. Considering normal distribution, student’s t-tests or ANOVA and Tukey’s multiple comparison tests were initially conducted to define notable difference at *P*-values<0.05 microbiological counts (SPSS 16 IBM Co. USA)

## Results

***MTT assay***

**Figure 1:**
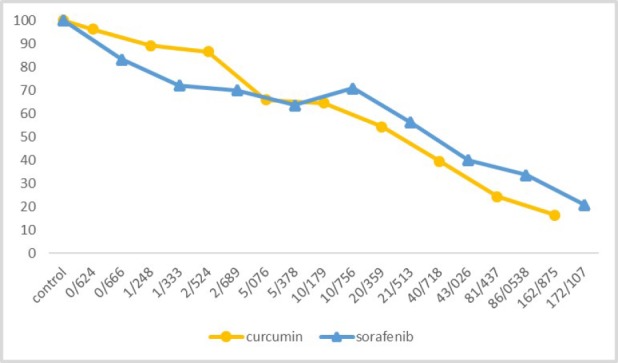
Nanomicelle curcumin and sorafenib viability

After triplicate MTT assay the results showed no changes in viability in 0.23 mg/ml concentration, but after increasing amount of nanomicelle curcumin concentration gradually, the viability decreased as well as in 60 mg/ml, the percentage has reached 20% (Figure 1). Using PRISM software (version 5), IC_50_ has been calculated for nanomicelle in which 4.14 has been showed as IC_50_. On the other hand; following the increasing of sorafenib concentration, the viability will decrease linearly, as well as in 80 mg/ml the percentage reaches its lower level. Using PRISM software (version 5), IC_50_ has been calculated for sorafenib in which 14 mg/ml has been showed as IC_50_. As samples have been studied in triplicates, the proportion of the percentage of viability to the control sample, which is 100%, has been calculated.

***RNA extraction***

To determine the RNA concentration and purity, the optical density of one of the extracted RNA samples (treated with nanomicelle curcumin) in 260/280 nm, calculated 1.92 by NanoDrop. The mean concentration of extracted RNA was 867.3 ng/µl. 

***Real-time PCR results***

After CT specified for each sample, ΔΔCT was calculated, and the gene expression ratio for each sample considered using the gene ratio law. The gene expression ratio (the ratio of cyclineD1 expression to GAPDH) of control sample calculated 1, because there was no treating.

The gene expression ratio showed the number of 289/315 while treating with sorafenib; this ratio was 128 while treating with nanomicelle curcumin and also this ratio was 152/365 while treating with nanomicelle curcumin and sorafenib combination. Based on the ANOVA statistical test, there were statistical differences between the three groups (*P*<0.001). Also based on the Tukey statistical test, there were statistical differences as well.

## Discussion

HCC is the most prevalent cancer among the primary liver tumors, also it is the third most common source of cancer-related mortality in the world. HCC is known as an aggressive carcinoma, and it is hard to diagnose also has limited therapeutic options. Due to the failure in sorafenib response in most patients and lack of alternative eﬀective therapeutic, to improve therapeutic efciency of sorafenib on HCC, it is vital to detect a new attitude (5). 

Investigation for controlled delivery of curcumin into the target tissues and organs has been an important issue for recent decade. Though, there are many researches on the advantages of curcumin, further research is needed for its clinical usage (17). Due to low bioavailability in free formulation, different strategies have been examined to improve curcumin bioavailability as nano-micelles and nanoparticles (18). In this study, expression of CCND1 gene has been shown 289.31, 128 and 152.36 for sorafenib, nanomicelle curcumin and SNC respectively which was statistically significant (*P*<0.001). 

**Figure 2:**
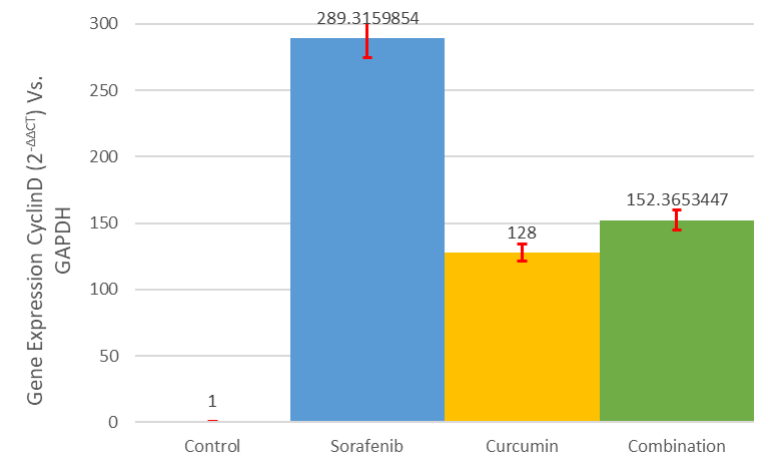
Gene expression status in control and treated samples

**Table 1:** The concentration of nanomicelle curcumin and sorafenib in MTT assay

	Concentration								
Nanomicelle Curcumin(mg/mL)	**0.23**	**0.46**	**0.93**	**1.87**	**3.75**	**7.5**	**15**	**30**	**60**
mmol/L	0.624	1.248	2.524	5.076	10.179	20.359	40.718	81.437	162.875
Sorafenib(mg∕mL)	0.31	0.62	1.25	2.5	5	10	20	40	80
mmol/L	0.666	1.333	2.689	5.378	10.756	21.513	43.026	86.0538	172.107

**Table 2:** Primers used in this study

Primers	forward	reverse
Cyclin D1	TGCACCACCAACTGCTTAGC	GGCATGGACTGTGGTCATGAG
GAPDH	GGATGCTGGAGGTCTGCGAGGAAC	GAGAGGAAGCGTGTGAGGCGGTAG

Our finding showed that treatment of HUH7 with nanomicelle curcumin IC_50_ dose and in combination with sorafenib might down-regulate CCND1 gene expression in compared to sorafenib alone. Cyclin D1 has been identified for its oncogenic activities and is a key regulator of cell cycle progression. It has been suggested that amplification and over-expression of the cyclin Dl gene play a role in multistep hepatocarcinogenesis, especially in the acceleration of tumor growth and the decrease in survival rate (19-21). 

Sorafenib is an oral multi-kinase inhibitor that suppresses tumor cell production by aiming Raf/MEK/ERK signaling at the level of Raf kinase and utilizes an antiangiogenic influence by aiming vascular endothelial development factor receptor -2/-3(VEGFR-2/-3), and platelet-derived growth factor receptor beta (PDGFR-) tyrosine kinases (22). 

In some studies, it has been shown that nanomicelle curcumin has a plant origin, in comparison to sorafenib which is a chemical drug, has better toxicity profile, economical, and the availability of raw materials for production. The safety of curcumin has been reported in many animal studies as well as human trials. Curcumin additionally down-regulated the mRNA and the protein expression of cyclin D1 and blocked transition of the cells from G1 to S phase (23, 24). 

Moreover, curcumin inhibits the proliferation of various tumor cell lines, and in most cells, this inhibition is related to the down-regulation of the expression of cyclin D1 protein (25). The cell cycle inhibitory effect in all human tumor cell lines, and also apoptosis in a subset of lines were tested. Comprehensive cell cycle analysis exposed that sorafenib can be grounds for a prolongation in the G1 phase. Mechanistic studies showed the influence of sorafenib on cyclin D1 and Rb expression (26). Amplification of the cyclin Dl gene was detected in 4 of the 30 (13%) HCCS from Taiwan (27). The combination of sorafenib and CDK inhibitors may improve the efficiency of sorafenib in hepatocellular carcinoma (28). Regarding the results of the gene expression in treated samples, the nanomicelle curcumin has been able to reduce the gene expression level of CCND1 alone and in combination with sorafenib. Curcumin could inhibit cyclin Dl gene in the other cell line (29). 

In treating the cell line with nanomicelle curcumin alone, the main effect seems to be a reduction in cyclin D1 expression, but when the curcumin is combined with sorafenib, in addition to the decrease of gene expression, apoptosis is induced. Accordingly, the toxicity of sorafenib and the reduction of gene expression related to the nanomicelle has been shown to double. According to our study, the assessment of nanomicelle curcumin on other genes in molecular pathways of HCC such as EGFR and VEGF is suggested. Thus, evaluation of cytotoxicity of nanomicelle curcumin combined with radiotherapy on HUH7 cell line along with animal studies is recommended. 

## Conclusion

Our study indicated that the foremost effect of treatment of the cell line with nanomicelle curcumin alone is down-regulation of cyclin D1. In addition to the decrease in the cyclin D1 expression, co-delivery of curcumin and sorafenib seems to induce the apoptotic process. By the same token, the effect of sorafenib cytotoxicity and CCND1 gene expression were increased twofold. The findings of this study support further investigation of using the co-delivery of sorafenib and curcumin as a new approach.
